# Supplementary material for: Estimates of protection levels against SARS-CoV-2 infection and severe COVID-19 in Germany before the 2022/2023 winter season: the IMMUNEBRIDGE project
Source: Infection. 2023 Aug 2;52(1):139–53. doi: 10.1007/s15010-023-02071-2 (PMC10811028; doi:10.1007/s15010-023-02071-2)
Supplement: Supplementary file 1 — Supplementary file1 (PDF 1800 KB) [file 15010_2023_2071_MOESM1_ESM.pdf]

Article title

Estimates of protection levels against SARS-CoV-2 infection and severe COVID-19 in Germany before the 2022/2023 winter season - the IMMUNEBRIDGE project

Journal
Infection

Authors
Berit Lange[§1,2], Veronika K Jaeger[§3], Manuela Harries[1], Viktoria Rücker[4], Hendrik Streeck[5,6], Sabine Blaschke[7], Astrid Petersmann[8,9], Nicole Toepfner[10], Matthias Nauck[9,11], Max J Hassenstein[1], Maren Dreier[12], Isabell Von Holt[12], Axel Budde[5,6], Antonia Bartz[3], Julia Ortmann[1], Marc-André Kurosinski[3], Reinhard Berner[10*], Max Borsche[13*], Gunnar Brandhorst[8*] Melanie Brinkmann[12*], Kathrin Budde[9*], Marek Deckena[14*], Geraldine Engels[15*], Marc Fenzlaff[9*], Christoph Härtel[15*], Olga Hovardovska[1*], Alexander Katalinic[16*], Katja Kehl[5,6*], Mirjam Kohls[4,17,18*], Stefan Krüger[19*], Wolfgang Lieb[20*] , Kristin M Meyer-Schlinkmann[14*], Tobias Pischon[21,22,23*], Daniel Rosenkranz[8*], Nicole Rübsamen[3*], Jan Rupp[24*], Christian Schäfer[9*], Mario Schattschneider[9*], Anne Schlegtendal[25*], Simon Schlinkert[19*], Lena Schmidbauer[4*], Kai Schulze-Wundling[5,6*], Stefan Störk[26*], Carsten Tiemann[14*], Henry Völzke[27*], Theresa Winter[9*], Christine Klein[13], Johannes Liese[15], Folke Brinkmann[25], Patrick F Ottensmeyer[5,6], Jens-Peter Reese[4], Peter Heuschmann[4,17,18], André Karch[&3]

Affiliations
[1] Department of Epidemiology, Helmholtz Centre for Infection Research (HZI), Braunschweig, Germany
[2] German Center for Infection Research (DZIF), TI BBD, Braunschweig, Germany
[3] Institute of Epidemiology and Social Medicine, University of Münster, Münster, Germany
[4] Institute of Clinical Epidemiology and Biometry, University Würzburg, Würzburg, Germany
[5] Institute of Virology, Medical Faculty, University of Bonn, Bonn, Germany
[6] German Center for Infection Research (DZIF), partner site Bonn-Cologne, Braunschweig, Germany
[7] Emergency Department, University Medical Center Goettingen, Goettingen, Germany
[8] Institute of Clinical Chemistry and Laboratory Medicine, University Medicine Oldenburg, Oldenburg, Germany
[9] Institute of Clinical Chemistry and Laboratory Medicine, University Medicine Greifswald, Greifswald, Germany
[10] Department of Pediatrics, University Hospital Carl Gustav Carus and Carl Gustav Carus Faculty of Medicine, TU Dresden, Dresden, Germany
[11] DZHK (German Centre for Cardiovascular Research), Partner Site Greifswald, University Medicine, Greifswald, Germany
[12] Institute for Epidemiology, Social Medicine and Health Systems Research, Hannover Medical School, Hannover, Germany
[13] Institute of Neurogenetics, University of Lübeck, Lübeck, Germany
[14] Labor Krone, Bad Salzuflen, Germany
[15] University Hospital Würzburg, Department of Pediatrics, Pediatric Infectiology, Würzburg, Germany
[16] Institute of Social Medicine and Epidemiology, University of Luebeck, Luebeck, Germany
[17] Clinical Trial Center, University Hospital Würzburg, Würzburg, Germany;
[18] Institute for Medical Data Science, University Hospital Würzburg, Würzburg, Germany
[19] dimap, das Institut für Markt- und Politikforschung GmbH, Germany
[20] Institute of Epidemiology, Christian-Albrechts-University of Kiel, Kiel, Germany
[21] Molecular Epidemiology Research Group, Max Delbrück Center for Molecular Medicine in the Helmholtz Association (MDC), Berlin, Germany
[22] Biobank Technology Platform, Max Delbrück Center for Molecular Medicine in the Helmholtz Association (MDC), Berlin, Germany
[23] Charité – Universitätsmedizin Berlin, Berlin, corporate member of Freie Universität Berlin and Humboldt-Universität zu Berlin, Berlin Germany
[24] Department of Infectious Diseases and Microbiology, University Hospital Schleswig-Holstein, German Center for Infection Research (DZIF), Partner Site Hamburg-Lübeck-Borstel-Riems, Lübeck, Germany
[25] University Children's Hospital, Ruhr University Bochum, Bochum, Germany

[26] Department of Clinical Research & Epidemiology, Comprehensive Heart Failure Center (CHFC), and Department of Internal Medicine I, University Hospital Würzburg, Würzburg, Germany
[27] Institute for Community Medicine, University Medicine Greifswald, Greifswald, Germany

*Members of the IMMUNEBRIDGE STUDY GROUP, listed in alphabetical order
[§]indicates shared first authorship
[&]indicates last authorship

Corresponding authors
Berit Lange (Berit.Lange@helmholtz-hzi.de) & André Karch (akarch@uni-muenster.de)

Supplementary material 1: Additional information on data collection and combined endpoint results

Figure S1: Combined endpoint on the proportion of exposures by survey month and age
Figure S2: Combined endpoint on the proportion of exposures by NUTS (age-standardised)
Figure S3: Combined endpoint on the proportion of exposures by federal states (age-standardised)
Figure S4: Combined endpoint on the proportion of exposures by NUTS (not age-standardised)
Figure S5: Combined endpoint on the proportion of exposures by federal states (not age-standardised)
Figure S6: Combined endpoint on the proportion of exposures: sensitivity analysis

Table S1: Description of the studies participating in IMMUNEBRIDGE
Table S2: Data collection instruments and infrastructures
Table S3: Vaccination status of participants stratified by age, sex, and pre-existing conditions and age-standardised
Table S4: Participants' self-reported first infections in 2020, 2021, and 2022 stratified by age, sex, and pre-existing conditions and age-standardised
Table S5: Combined endpoint of infection, vaccination, and humoral immunity as well as proportion of antibodies detected against the S-antigen (S-AK) and the N-antigen (N-AK) stratified by age, sex, and pre-existing conditions (not age-standardised)
Table S6: Vaccination status of participants stratified by age, sex, and pre-existing conditions (not age-standardised)
Table S7: Participants' self-reported first infections in 2020, 2021, and 2022 stratified by age, sex, and pre-existing conditions (not age-standardised)

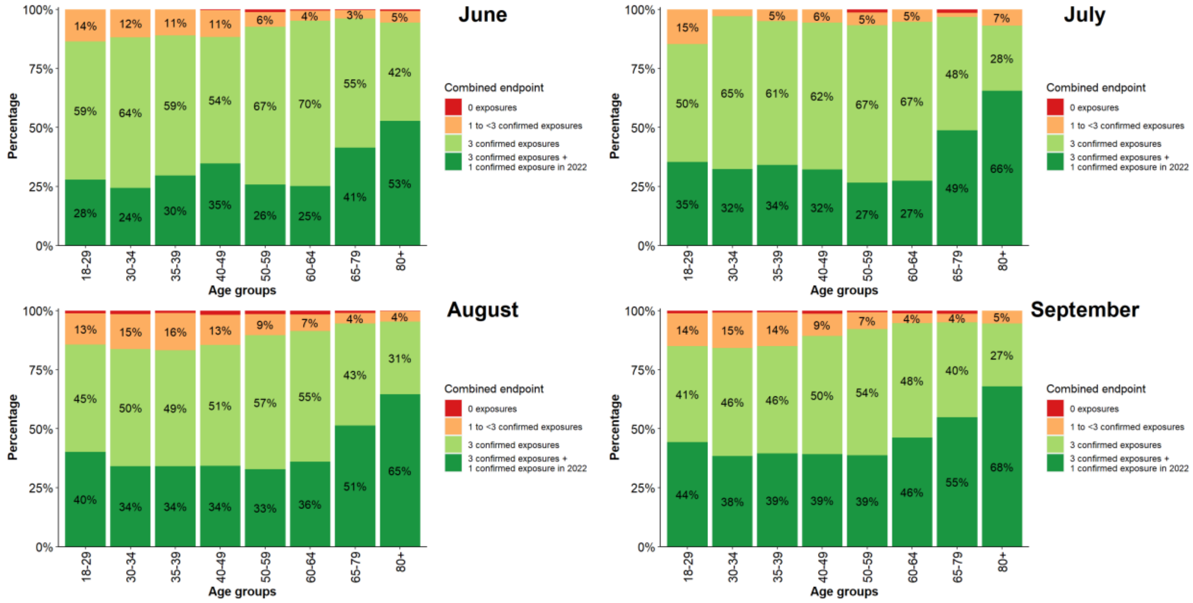

Supplement 1 Figure S1: Combined endpoint on the proportion of exposures through infection or vaccination with corresponding humoral immune response stratified by survey month and age (≥ 18 years); for the definition of endpoints see Table 1 in the main manuscript.

Regional analysis of the combined endpoints

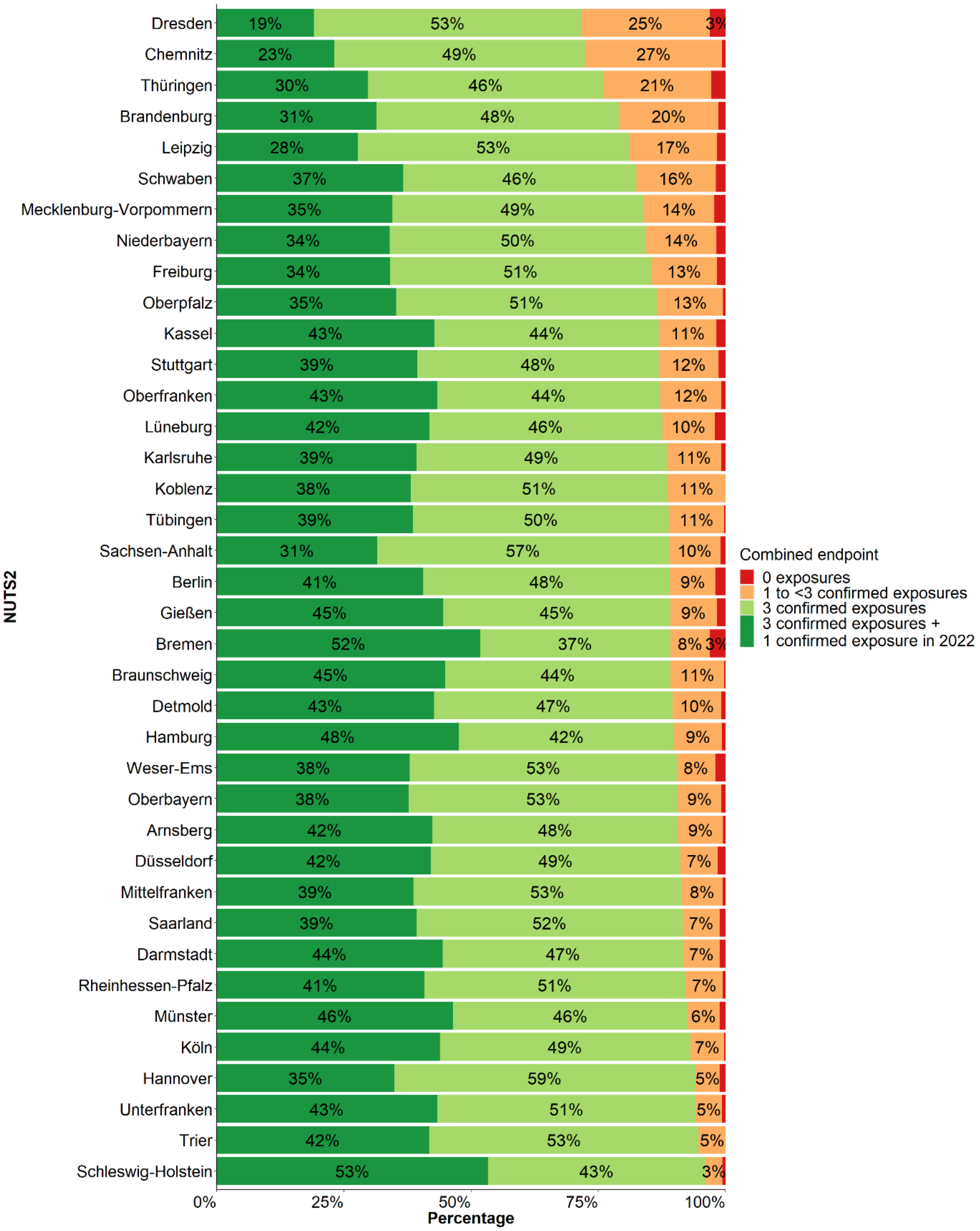

Supplement 1 Figure S2: Combined endpoint on the proportion of exposures through infection or vaccination with corresponding humoral immune response stratified by NUTS-2 and age-standardised (≥18 years).

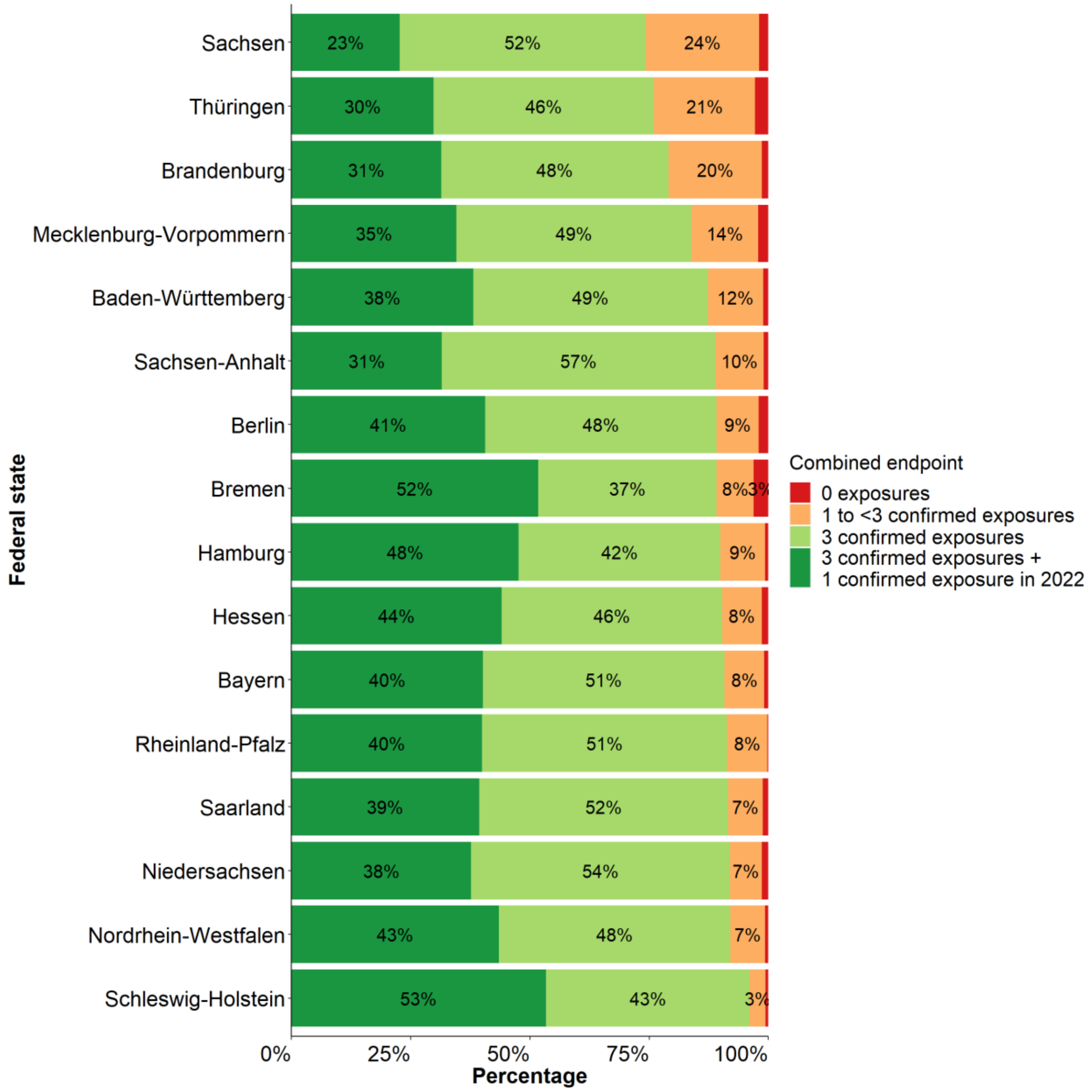

Supplement 1 Figure S3: Combined endpoint on the proportion of exposures through infection or vaccination with corresponding humoral immune response stratified by federal state and age-standardised (≥18 years).

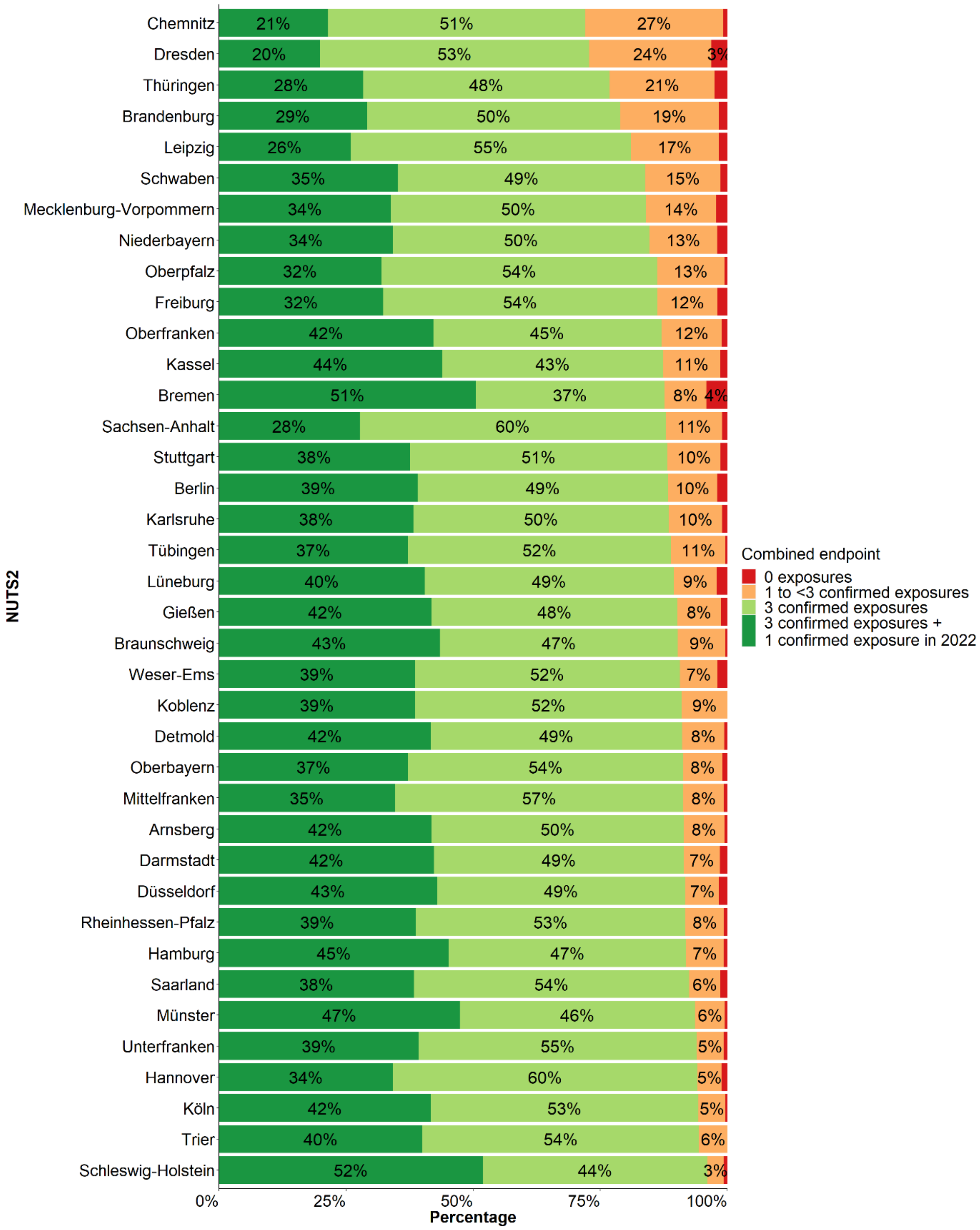

Supplement 1 Figure S4: Combined endpoint on the proportion of exposures through infection or vaccination with corresponding humoral immune response stratified by NUTS-2 (not age-standardised; ≥18 years).

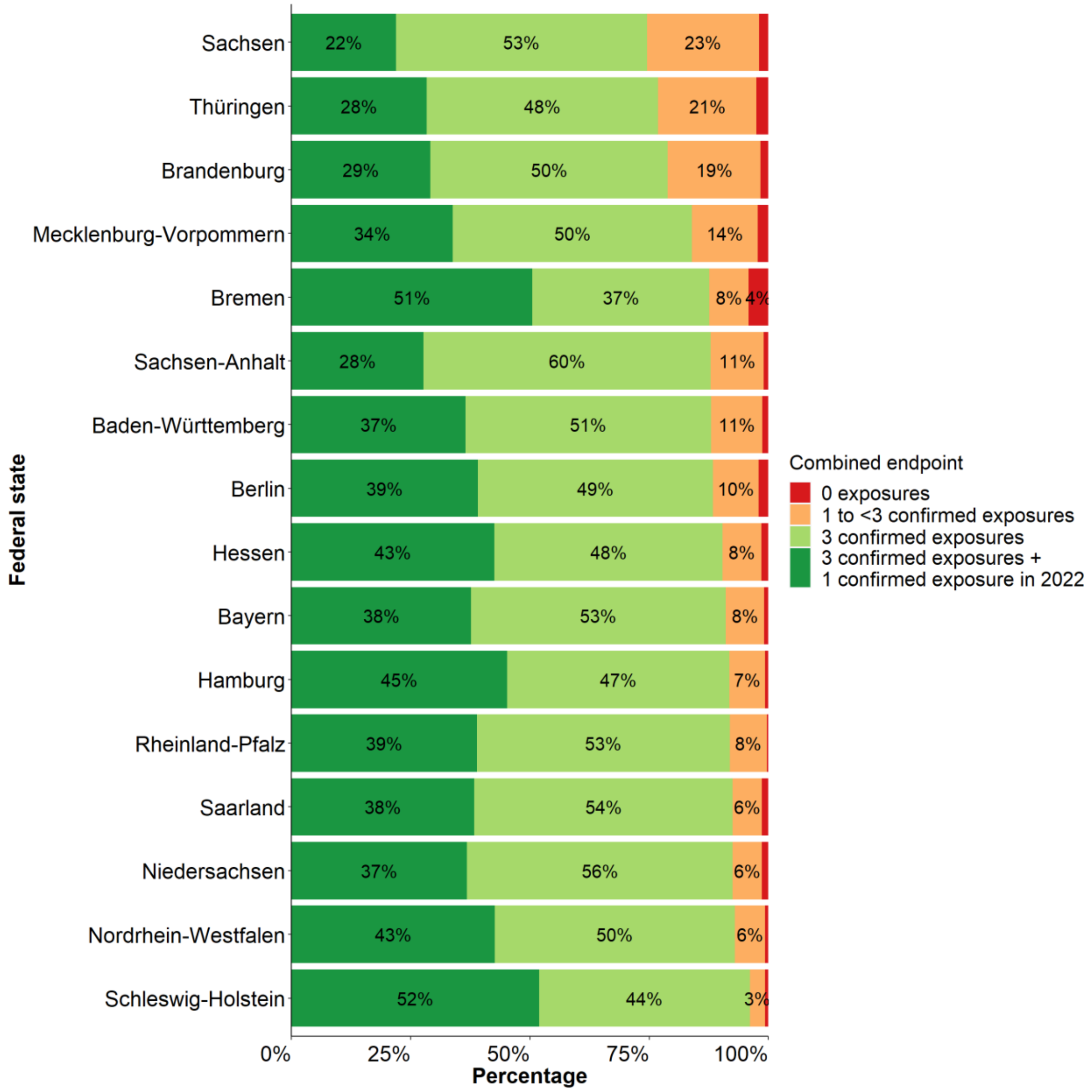

Supplement 1 Figure S5: Combined endpoint on the proportion of exposures through infection or vaccination with corresponding humoral immune response stratified by federal state (not age-standardised; ≥18 years).

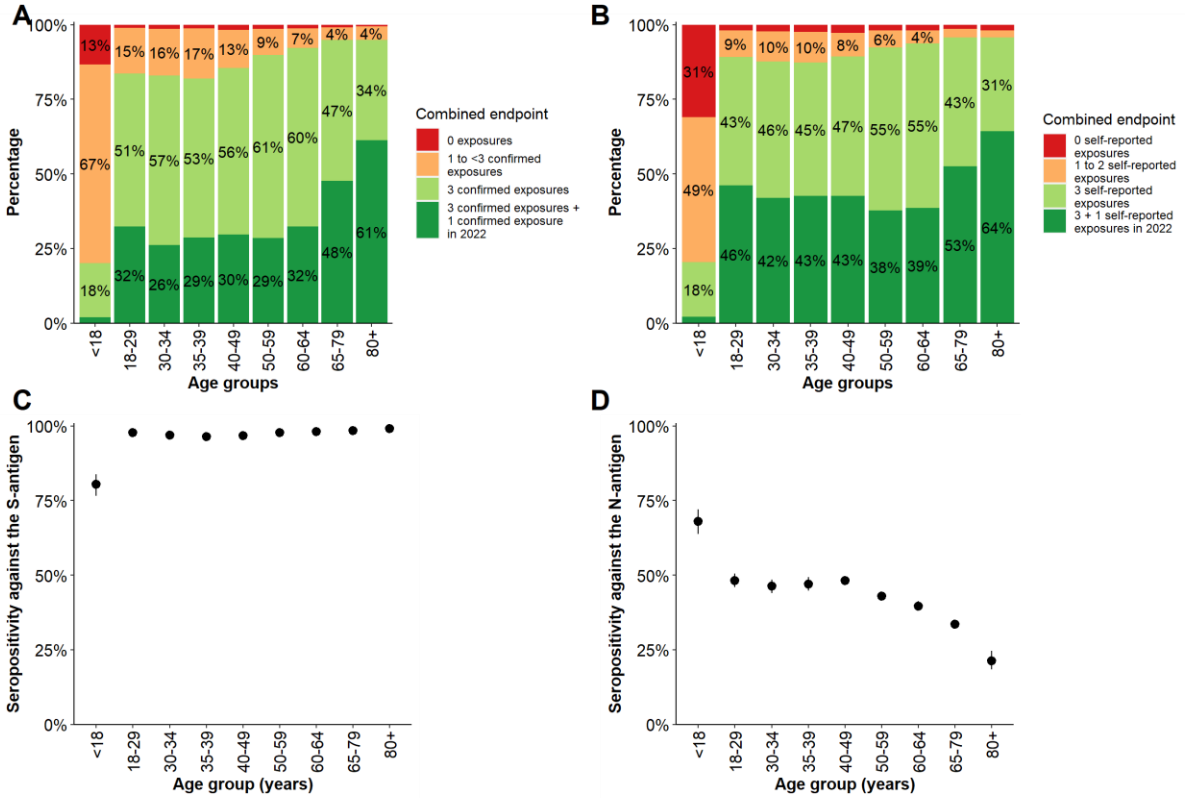

Supplement 1 Figure S6: Sensitivity analysis treating borderline N-antigen findings of the GUIDE study as seronegative (A) The combined endpoint on the proportion of exposures by infection or vaccination with corresponding humoral immune response, stratified by age (B) the combined endpoint of the proportion of exposures by infection or vaccination regardless of a humoral immune response, stratified by age; (C) seropositivity against the S-antigen and (D) against the N-antigen by age, with 95% confidence intervals.

Participants from IMMUNEBRIDGE_ED were excluded from this analysis. The combined endpoint could not be formed for 12,185 participants due to missing information from the participants from NAKO (n=10,595) and for additional 1,590 participants from the other cohorts. Vaccination recommendations for children differed from those for adults, recommendations of > 1 dose only currently exist for those > 11 years old.

Supplement 1 Table S1: Description of the studies participating in IMMUNEBRIDGE

	Financing of data collection*	Description of the cohort	Cohort type	Region	N	Existing lab parameters	Existing data items	Restrictions
**Population-based cohorts (18 years and older)**								
NAKO	Federal Ministry of Education and Research, federal statues, Helmholtz Association	10.1007/s10654-014-9890-7	Population-based; recruitment 2014-2019 via population registration offices	18 locations nationwide	10595	N-AK, S-AK	Age, gender	Age at baseline 20-69 years
MuSPAD	Helmholtz Association, RESPINOW (Federal Ministry of Education and Research), own funds	10.3238/arztebl.m2021.0364; https://hzi-c19-antikoerperstudie.de/teilnehmerunterlagen/	Population-based; recruitment 2020 via population registration offices	Magdeburg	1046	N-AK, S-AK, S-IGRA	MDS** present, previous findings from 2 surveys	≥18
MuSPAD	Helmholtz Association, RESPINOW (Federal Ministry of Education and Research), own funds	10.3238/arztebl.m2021.0364; https://hzi-c19-antikoerperstudie.de/teilnehmerunterlagen/	Population-based; recruitment 2020 via population registration offices	Aachen	870	N-AK, S-AK, S-IGRA	MDS** present, previous findings from 2 surveys	≥18
MuSPAD	Helmholtz Association, IMMUNEBRIDGE (Federal Ministry of Education and Research), own funds	10.3238/arztebl.m2021.0364; https://hzi-c19-antikoerperstudie.de/teilnehmerunterlagen/	Population-based; recruitment 2020 via population registration offices	Hanover	1116	N-AK, S-AK	MDS** present, previous findings from 1 survey	≥18
STAAB	Federal Ministry of Education and Research, Bavarian State Ministry of Science and the Arts	10.1177/2047487316680693	Population-based; recruitment baseline 2013-2017 via population registration offices	Würzburg	1711	N-AK, S-AK	MDS** present, previous findings from 2 surveys	Age at baseline 30-79 years
ELISA	IMMUNEBRIDGE (Federal Ministry of Education and Research)	https://www.ncbi.nlm.nih.gov/pmc/articles/PMC9012459/pdf/sciadv.abm5016.pdf https://science.org/doi/10.1126/sciadv.abm5016	Population-based; recruitment 2020 via population registration offices	Lübeck	1389	N-AK, S-AK	MDS** present	≥18
**Cross-sectional studies on children and populations with risk factors for a severe course of SARS-CoV-2 infection**								
Dresden paedSAXCOVID	Funding by the Free State of Saxony "paedSaxCoviDD	10.1136/bmjpo-2021-001036, 10.1007/s15010-022-01824-9	School-based	Dresden	91	N-AK, S-AK	MDS** present	12-17
Würzburg Wü-KITa-CoV	Bavarian State Ministry of Health and Care/ State Office for Health and Food Safety	https://jamanetwork.com/journals/jamanetworkopen/fullarticle/2787578 https://jamanetwork.com/journals/jamanetworkopen/fullarticle/2796275	Based on day care centres	Würzburg	275	N-AK, S-AK	MDS** present	1-6
Bochum CorKID	IMMUNEBRIDGE (Federal Ministry of Education and Research)	10.3390/idr13040088 10.1001/jamanetworkopen.2021.42057	Participating paediatric practices, recruitment July-August 2022	Ruhr area	189	N-AK, S-AK	MDS** present	1-17
IMMUNEBRIDGE_ED	Own resources	-	Patients from the emergency room	Göttingen	423	N-AK, S-AK	MDS** present	≥18
**Cross-sectional study of the general population in Germany (UK Bonn)**								
GUIDE (Payback + CATI)	IMMUNEBRIDGE (Federal Ministry of Education and Research)	https://www.drks.de/drks_web/navigate.do?navigationId=trial.HTML&TRIAL_ID=DRKS00029693	Random draw from participants of the Payback Panel (online) + CATI survey (telephone) of persons older than 65 years of age	Germany-wide	15932	N-AK, S-AK (DBS)	MDS** present	≥18
**Total**					33637			

*In addition, the central laboratory analysis in Oldenburg and Greifswald in the population cohort studies was financed via the IMMUNEBRIDGE project; **MDS = Minimal data set

Supplement 1 Table S2: Data collection instruments and infrastructures

Characteristics needed	Definition/ Conditions	Survey infrastructures needed	N IMMUNEBRIDGE studies meeting this characteristic	(Network) infrastructures to ensure this	How Immunebridge met this characteristic
Population-based design		Ability to work with population registers	5 (ELISA,GUIDE, MuSPAD, NAKO, STAAB)	-	-
Longitudinal design	Scheduled follow-ups or ability to recontact and reinvite participants		4 (ELISA, MuSPAD, NAKO, STAAB)	-	-
Supra-regional design	Within surveys (not always needed)	Study centre ability in several locations	4 (GUIDE, MuSPAD, NAKO, STAAB)	Geographic depths of the network either ensured or able to pull in additional sources on short notice	Set up additional cross sectional survey (GUIDE) to gain geographic depth
Speed	Survey preparation within 1-2 months	Logistics need to be set up to ensure short preparation time	3 (MuSPAD, NAKO, STAAB)	Overview of studies and their potential preparation times needs to be available	Close consultation with the individual cohorts from the planning phase onwards
	Data linkage within 1-2 weeks	Ability to integrate minimal data sets quickly into current surveys	8 (all)	Data linkage capacity and tool needs to be available	Relevant staff capacities of several research institutions were planned for this from the beginning
	Data analysis of aggregate estimates within 1-2 weeks		8 (all)	Data analysis capacity needs to be available	Relevant staff capacities of several research institutions were planned for this from the beginning
	Ensuring capacity to be speedy in set up of surveys and network proceedings by regular use (min 1/year)	Regular use of cohort for different purposes and pathogens	4 (ELISA, MuSPAD, NAKO, STAAB)	Regular (min 1/year) use of network	Part of the surveys performed were already as part of the regular use of epidemic panels (e.g. MuSPAD within RESPINOW)
Adaptability	Of the survey to different pathogens (not needed in IB)	Different sampling methods need to be available (e.g., blood, swab, stool)	3 (MuSPAD, NAKO, STAAB)	Network needs to be able to ensure that different sampling methods across studies are available	Did not need to be met, as only one pathogen was assessed
Linkage of survey and sampling across the network	Comparability of used biomarker assays	Potential to adapt to central laboratory infrastructure	9 (all)	Central laboratory infrastructures, including IT linkage to ensure comparability	Central laboratory structure experience from NAKO and NUM-NAPKON was used here
	Quick set-up of minimal data sets	Generic minimal data sets implemented in individual studies	9 (all)	Generic minimal data sets available within the network	A minimal data set was set up at the start of the project
Data linkage with other relevant sources	Surveillance data		perspective	-	-
	Hospital data		perspective	-	-
	Ability to link to clinical cohorts		perspective	Either the network comprises clinical cohorts or has close collaboration with some	A clinical cohort as comparison (emergency department) was established

Supplement 1 Table S3: Vaccination status of participants stratified by age, sex, and pre-existing conditions and age-standardised. Participants from IMMUNEBRIDGE_ED were excluded from this analysis. Information on the vaccination status of NAKO participants (n=10,595) and 262 participants from the other cohorts is unknown and are therefore not included in this analysis. (LCI: lower 95% confidence interval limit; UCI: upper 95% confidence interval limit)

		No vaccination			First dose			Second dose			Third dose			Fourth dose		
	N	Share (%)	LCI	UCI	Share (%)	LCI	UCI	Share (%)	LCI	UCI	Share (%)	LCI	UCI	Share (%)	LCI	UCI
**Total**	22225	14.0	13.4	14.7	1.4	1.1	1.7	11.5	10.8	12.2	58.8	58.1	59.5	14.3	13.9	14.7
**Age (years)**																
1-17	534	61.8	57.5	65.9	3.4	2.1	5.4	26.6	22.9	30.6	8.2	6.1	11.0	0	0	8.8
18-29	1617	5.3	4.3	6.6	1.4	0.9	2.1	14.7	13.0	16.5	75.7	73.5	77.8	3.0	2.2	4.0
30-34	1430	6.8	5.6	8.2	1.3	0.8	2.0	13.0	11.3	14.9	75.9	73.6	78.1	3.0	2.2	4.1
35-39	1478	7.2	5.9	8.6	1.4	0.9	2.1	13.3	11.6	15.1	73.4	71.1	75.6	4.8	3.8	6.1
40-49	3138	6.7	5.9	7.7	1.4	1.0	1.9	9.6	8.6	10.7	76.1	74.6	77.6	6.2	5.4	7.1
50-59	5158	4.2	3.7	4.8	0.8	0.6	1.1	7.3	6.6	8.0	78.0	76.8	79.1	9.8	9.0	10.6
60-64	2364	3.1	2.5	3.9	0.7	0.4	1.1	5.6	4.7	6.6	74.3	72.5	76.0	16.3	14.9	17.9
65-79	5739	2.0	1.7	2.4	0.5	0.3	0.7	3.4	2.9	3.9	56.5	55.3	57.8	37.6	36.4	38.9
80 and older	767	2.1	1.2	3.4	0.8	0.3	1.8	2.5	1.5	3.9	34.7	31.3	38.2	60.0	56.4	63.5
**Sex**																
Female	11736	13.4	12.3	14.6	1.5	1.1	2.0	11.5	10.5	12.5	60.4	59.2	61.5	13.2	12.6	13.8
Male	10488	14.7	13.5	16.0	1.2	0.9	1.6	11.5	10.4	12.5	57.1	55.9	58.3	15.6	14.9	16.3
**Comorbidities***																
Cancer (current or treated in the last year)	1092	2.2	1.2	3.1	0.4	0.1	0.8	4.8	3.4	6.2	55.6	52.1	59.0	37.1	33.7	40.4
Cardiovascular disease	2014	1.8	1.3	2.4	0.5	0.1	0.8	3.8	2.9	4.8	52.0	49.4	54.5	41.9	39.4	44.5
Diabetes	2028	3.2	2.4	4.1	0.5	0.2	0.8	4.8	3.7	5.8	55.8	53.4	58.3	35.7	33.3	38.1
Hypertension	7082	2.6	2.2	3.1	0.7	0.5	0.9	5.1	4.5	5.6	61.6	60.4	62.9	30.0	28.8	31.2
Immunosuppression	434	1.4	0.3	2.5	0.6	0.1	1.3	4.9	2.7	7.1	57.9	52.8	63.1	35.2	30.2	40.2
Lung disease	2520	3.2	2.5	3.9	0.9	0.5	1.2	8.6	7.3	9.8	64.1	62.0	66.2	23.2	21.4	25.1

*only in the ≥ 18years

Supplement 1 Table S4: Proportion of participants' self-reported first infections in 2020, 2021, and 2022 stratified by age, sex, and pre-existing conditions and age-standardised. Participants from IMMUNEBRIDGE_ED were excluded from this analysis. Information on the date of self-reported infections of NAKO participants (n=10,595) and 365 participants from the other cohorts is unknown; these are therefore not included in this analysis. (LCI: lower 95% confidence interval limit; UCI: upper 95% confidence interval limit)

		No reported infection			First reported infection 2020			First reported infection 2021			First reported infection 2022		
	N	Share (%)	LCI	UCI	Share (%)	LCI	UCI	Share (%)	LCI	UCI	Share (%)	LCI	UCI
**Total**	22122	58.6	57.7	59.5	2.1	1.9	2.3	4.5	4.1	4.9	34.8	33.9	35.7
**Age (years)**													
1-17	516	54.3	49.9	58.6	1.2	0.5	2.6	3.5	2.1	5.6	41.1	36.8	45.5
18-29	1616	41.8	39.4	44.3	3.3	2.5	4.4	6.4	5.3	7.7	48.5	46.0	50.9
30-34	1429	45.6	43.0	48.3	3.2	2.3	4.2	6.0	4.9	7.4	45.2	42.6	47.8
35-39	1476	46.5	43.9	49.1	2.3	1.6	3.2	6.4	5.3	7.8	44.8	42.2	47.4
40-49	3125	49.2	47.4	51.0	2.7	2.1	3.3	5.9	5.1	6.8	42.3	40.5	44.0
50-59	5118	62.1	60.8	63.4	2.2	1.8	2.6	4.5	4.0	5.1	31.2	30.0	32.5
60-64	2356	69.1	67.1	70.9	2.1	1.6	2.8	3.4	2.8	4.3	25.4	23.7	27.2
65-79	5717	76.1	74.9	77.2	1.4	1.2	1.8	2.6	2.2	3.1	19.9	18.9	21.0
80 and older	769	83.1	80.2	85.6	1.2	0.6	2.3	3.0	2.0	4.5	12.7	10.5	15.4
**Sex**													
female	11670	58.5	57.2	59.8	1.9	1.6	2.2	4.2	3.7	4.7	35.5	34.2	36.7
male	10451	58.7	57.3	60.0	2.3	2.0	2.7	4.9	4.3	5.5	34.1	32.8	35.5
**Antibodies**													
N-AB positive	9084	30.0	28.6	31.5	3.0	2.6	3.5	7.0	6.3	7.7	60.0	58.5	61.5
N-AB negative	11646	85.6	84.8	86.4	1.3	1.1	1.5	2.2	1.9	2.5	11.0	10.2	11.7
S-AB positive	20038	57.7	56.8	58.7	2.2	1.9	2.5	4.7	4.3	5.1	35.4	34.5	36.3
S-AB negative	678	72.4	67.6	77.2	0.8	0.2	1.3	1.8	1.0	2.5	25.1	20.4	29.8
**Comorbidities***													
Cancer (current or treated in the last year)	1091	70.9	67.9	73.9	1.8	1.1	2.5	3.5	2.3	4.7	23.8	21.0	26.7
Cardiovascular disease	2001	74.1	71.9	76.3	2.2	1.5	2.9	3.1	2.2	4.0	20.6	18.6	22.6
Diabetes	2028	72.4	70.3	74.5	1.6	1.0	2.3	5.1	4.0	6.3	20.9	19.0	22.7
Hypertension	7055	69.1	68.0	70.3	2.0	1.7	2.4	4.1	3.6	4.7	24.7	23.6	25.8
Immunosuppression	429	71.4	66.9	76.0	2.1	0.8	3.3	3.2	1.5	4.9	23.3	19.1	27.6
Lung disease	2507	61.5	59.4	63.6	2.9	2.1	3.7	4.2	3.4	5.1	31.4	29.4	33.4

*only in the ≥ 18years

Supplement 1 Table S4: Combined endpoint of infection, vaccination, and humoral immunity as well as proportion of antibodies detected against the S-antigen (S-AK) and the N-antigen (N-AK) stratified by age, sex, and pre-existing conditions. The results are not age-standardised. Participants from IMMUNEBRIDGE_ED were excluded from this analysis as were participants with missing age information. The combined endpoint could not be formed for 12,185 participants due to missing information from the participants from NAKO (n=10,595) and for additional 1,590 participants from the other cohorts. (LCI: lower 95% confidence interval limit; UCI: upper 95% confidence interval limit).

	Combined endpoint													Antibodies							
		Three exposures + infection or vaccination in 2022			Three exposures with immunocorrelates			Three exposures without immunocorrelates or less than three exposures (min. 1 exposure/ immunocorrelate)			No immunocorrelates and no infection or vaccination			S-AK				N-AK			
	N	Share (%)	LCI	UCI	Share (%)	LCI	UCI	Share (%)	LCI	UCI	Share (%)	LCI	UCI	N	Share (%)	LCI	UCI	N	Share (%)	LCI	UCI
**Total**	20912	38.3	37.6	38.9	50.3	49.6	50.9	10.0	9.6	10.4	1.4	1.3	1.6	31566	97.4	97.3	97.6	31580	47.8	47.3	48.3
**Age (years)**																					
1-17	511	2.0	1.0	3.7	18.2	15.0	21.9	66.5	62.2	70.6	13.3	10.5	16.6	506	80.4	76.7	83.7	516	68.0	63.8	72.0
18-29	1522	39.7	37.2	42.2	45.9	43.3	48.4	13.4	11.8	15.2	1.1	0.6	1.7	1807	97.7	96.8	98.3	1809	59.7	57.3	61.9
30-34	1361	33.7	31.2	36.2	50.8	48.2	53.5	14.3	12.5	16.3	1.2	0.7	1.9	1914	97.0	96.1	97.7	1914	56.8	54.6	59.1
35-39	1402	34.4	31.9	36.9	49.9	47.3	52.6	14.8	13.0	16.8	0.9	0.5	1.6	1910	96.4	95.5	97.2	1910	56.8	54.5	59.0
40-49	2994	34.8	33.1	36.6	51.7	49.9	53.5	12.0	10.9	13.3	1.4	1.1	1.9	4586	96.8	96.2	97.3	4586	56.0	54.5	57.4
50-59	4906	32.1	30.8	33.4	58.4	57.0	59.8	8.2	7.5	9.0	1.2	1.0	1.6	8112	97.7	97.3	98.0	8112	48.4	47.3	49.4
60-64	2246	34.8	32.8	36.8	57.9	55.8	59.9	6.1	5.2	7.2	1.2	0.8	1.8	3728	98.0	97.5	98.5	3729	43.9	42.3	45.5
65-79	5300	49.7	48.4	51.1	45.2	43.9	46.6	4.1	3.6	4.7	0.9	0.7	1.2	8310	98.5	98.2	98.7	8311	38.1	37.1	39.2
80 and older	670	62.5	58.7	66.2	32.5	29.0	36.3	4.6	3.2	6.6	0.3	0.1	1.2	693	99.1	98.0	99.6	693	28.3	25.0	31.8
**Sex**																					
Female	11123	37.9	37.0	38.8	50.7	49.8	51.6	9.9	9.3	10.4	1.5	1.3	1.7	16541	97.4	97.1	97.6	16550	46.7	45.9	47.4
Male	9788	38.7	37.7	39.6	49.8	48.8	50.8	10.2	9.6	10.8	1.3	1.1	1.5	14929	97.5	97.2	97.7	14934	49.0	48.2	49.8
**Comorbidities***																					
Cancer (current or treated in the last year)	1004	49.2	46.1	52.3	44.4	41.4	47.5	5.5	4.1	6.9	0.9	0.3	1.5	1006	97.5	96.6	98.5	1006	37.1	34.1	40.1
Cardiovascular disease	1885	50.8	48.5	53.0	43.6	41.4	45.8	4.9	4.0	5.9	0.7	0.3	1.1	1949	98.3	97.7	98.9	1949	37.5	35.4	39.7
Diabetes	1879	45.8	43.6	48.1	47.3	45.0	49.5	5.7	4.6	6.7	1.2	0.7	1.7	1904	97.7	97.1	98.4	1905	34.1	31.9	36.2
Hypertension	6621	44.6	43.4	45.8	49.4	48.2	50.6	5.3	4.7	5.8	0.8	0.6	1.0	6729	98.1	97.8	98.5	6730	38.9	37.8	40.1
Immunosuppression	414	48.8	44.0	53.6	46.4	41.6	51.2	4.3	2.4	6.3	0.5	0.0	1.2	415	98.8	97.7	99.8	415	36.9	32.2	41.5
Lung disease	2364	43.0	41.0	45.0	47.6	45.6	49.6	8.5	7.3	9.6	0.9	0.5	1.3	2393	97.5	96.9	98.2	2393	41.0	39.0	42.9
**First infections**																					
No infection	12790	22.7	22.0	23.4	68.5	67.7	69.2	7.0	6.6	7.4	1.8	1.6	2.0	12751	96.6	96.3	96.9	12762	21.4	20.7	22.1
First infection 2020	444	36.3	31.8	40.8	43.2	38.5	47.8	20.5	16.8	24.3	-	-	-	443	98.0	96.7	99.3	443	65.5	61.0	69.9
First infection 2021	921	33.8	30.7	36.9	38.1	34.9	41.3	28.1	25.2	31.1	-	-	-	912	97.5	96.5	98.5	912	71.1	68.1	74.0
First infection 2022	6637	72.5	71.4	73.6	19.3	18.3	20.3	8.2	7.5	8.9	-	-	-	6613	96.8	96.4	97.3	6616	81.8	80.9	82.7
**Vaccinations**																					
No vaccination	1203	0.1	0.0	0.3	0.3	0.0	0.7	74.1	71.3	77.0	25.4	22.6	28.3	1173	49.0	46.2	51.8	1181	61.4	58.6	64.2
One vaccination	208	0.0	0.0	0.0	12.6	7.9	17.3	87.4	82.7	92.1	-	-	-	197	92.4	88.7	96.1	197	64.5	57.8	71.2
Two vaccinations	1715	9.0	7.6	10.4	35.1	32.8	37.4	55.9	53.4	58.3	-	-	-	1664	98.8	98.3	99.3	1667	55.4	53.0	57.7
Three vaccinations	14231	31.1	30.3	31.8	68.7	67.9	69.4	0.3	0.2	0.3	-	-	-	14231	99.7	99.7	99.8	14234	45.0	44.2	45.9
Four vaccinations	3554	97.0	96.4	97.5	2.7	2.2	3.2	0.3	0.1	0.5	-	-	-	3554	99.7	99.5	99.9	3554	28.0	26.6	29.5

*only in ages ≥ 18years; S-AK: antibodies against S-antigen; N-AK: antibodies against nucleocapsid antigen

Supplement 1 Table S5: Vaccination status of participants stratified by age, sex, and pre-existing conditions. The results are not age-standardised. Participants from IMMUNEBRIDGE_ED were excluded from this analysis as were participants with missing age information. Information on the vaccination status of NAKO participants (n=10,595) and 262 participants from the other cohorts is unknown and are therefore not included in this analysis. (LCI: lower 95% confidence interval limit; UCI: upper 95% confidence interval limit)

		No vaccination			First dose			Second dose			Third dose			Fourth dose		
	N	Share (%)	LCI	UCI	Share (%)	LCI	UCI	Share (%)	LCI	UCI	Share (%)	LCI	UCI	Share (%)	LCI	UCI
**Total**	22225	5.6	5.4	5.9	1.0	0.8	1.1	8.0	7.7	8.4	68.0	67.4	68.6	17.4	16.9	17.8
**Age (years)**																
1-17	534	61.8	57.5	65.9	3.4	2.1	5.4	26.6	22.9	30.6	8.2	6.1	11.0	0	0	8.8
18-29	1617	5.3	4.3	6.6	1.4	0.9	2.1	14.7	13.0	16.5	75.7	73.5	77.8	3.0	2.2	4.0
30-34	1430	6.8	5.6	8.2	1.3	0.8	2.0	13.0	11.3	14.9	75.9	73.6	78.1	3.0	2.2	4.1
35-39	1478	7.2	5.9	8.6	1.4	0.9	2.1	13.3	11.6	15.1	73.4	71.1	75.6	4.8	3.8	6.1
40-49	3138	6.7	5.9	7.7	1.4	1.0	1.9	9.6	8.6	10.7	76.1	74.6	77.6	6.2	5.4	7.1
50-59	5158	4.2	3.7	4.8	0.8	0.6	1.1	7.3	6.6	8.0	78.0	76.8	79.1	9.8	9.0	10.6
60-64	2364	3.1	2.5	3.9	0.7	0.4	1.1	5.6	4.7	6.6	74.3	72.5	76.0	16.3	14.9	17.9
65-79	5739	2.0	1.7	2.4	0.5	0.3	0.7	3.4	2.9	3.9	56.5	55.3	57.8	37.6	36.4	38.9
80 and older	767	2.1	1.2	3.4	0.8	0.3	1.8	2.5	1.5	3.9	34.7	31.3	38.2	60.0	56.4	63.5
**Sex**																
Female	11736	5.8	5.4	6.2	1.0	0.8	1.2	7.9	7.4	8.4	69.0	68.2	69.9	16.3	15.7	17.0
Male	10488	5.5	5.1	5.9	0.9	0.7	1.1	8.2	7.6	8.7	66.9	66.0	67.8	18.6	17.9	19.3
**Comorbidities***																
Cancer (current or treated in the last year)	1092	2.2	1.3	3.1	0.6	0.1	1.0	5.0	3.7	6.2	57.8	54.9	60.7	34.5	31.7	37.3
Cardiovascular disease	2014	2.1	1.5	2.7	0.5	0.2	0.8	4.0	3.2	4.9	56.3	54.2	58.5	37.1	35.0	39.2
Diabetes	2028	3.0	2.3	3.8	0.6	0.3	0.9	4.4	3.5	5.3	58.2	56.1	60.3	33.8	31.8	35.9
Hypertension	7082	2.5	2.2	2.9	0.6	0.4	0.8	5.0	4.5	5.5	64.4	63.3	65.5	27.5	26.5	28.5
Immunosuppression	434	1.4	0.3	2.5	0.7	-0.1	1.5	4.6	2.6	6.6	58.3	53.7	62.9	35.0	30.5	39.5
Lung disease	2520	3.2	2.5	3.9	1.0	0.6	1.3	7.8	6.7	8.8	64.0	62.1	65.9	24.1	22.4	25.7

*only in the ≥ 18years

Supplement 1 Table S6: Proportion of participants' self-reported first infections in 2020, 2021, and 2022 stratified by age, sex, and pre-existing conditions. The results are not age-standardised. Participants from IMMUNEBRIDGE_ED were excluded from this analysis as were participants with missing age information. Information on the date of self-reported infections of NAKO participants (n=10,595) and 365 participants from the other cohorts is unknown; these are therefore not included in this analysis. (LCI: lower 95% confidence interval limit; UCI: upper 95% confidence interval limit)

		No reported infection			First reported infection 2020			First reported infection 2021			First reported infection 2022		
	N	Share (%)	LCI	UCI	Share (%)	LCI	UCI	Share (%)	LCI	UCI	Share (%)	LCI	UCI
**Total**	22122	61.6	61.0	62.2	2.1	1.9	2.3	4.4	4.1	4.7	31.9	31.3	32.5
**Age (years)**													
1-17	516	54.3	49.9	58.6	1.2	0.5	2.6	3.5	2.1	5.6	41.1	36.8	45.5
18-29	1616	41.8	39.4	44.3	3.3	2.5	4.4	6.4	5.3	7.7	48.5	46.0	50.9
30-34	1429	45.6	43.0	48.3	3.2	2.3	4.2	6.0	4.9	7.4	45.2	42.6	47.8
35-39	1476	46.5	43.9	49.1	2.3	1.6	3.2	6.4	5.3	7.8	44.8	42.2	47.4
40-49	3125	49.2	47.4	51.0	2.7	2.1	3.3	5.9	5.1	6.8	42.3	40.5	44.0
50-59	5118	62.1	60.8	63.4	2.2	1.8	2.6	4.5	4.0	5.1	31.2	30.0	32.5
60-64	2356	69.1	67.1	70.9	2.1	1.6	2.8	3.4	2.8	4.3	25.4	23.7	27.2
65-79	5717	76.1	74.9	77.2	1.4	1.2	1.8	2.6	2.2	3.1	19.9	18.9	21.0
80 and older	769	83.1	80.2	85.6	1.2	0.6	2.3	3.0	2.0	4.5	12.7	10.5	15.4
**Sex**													
female	11670	61.8	60.9	62.6	1.9	1.7	2.1	4.1	3.7	4.4	32.3	31.4	33.1
male	10451	61.4	60.5	62.3	2.4	2.1	2.7	4.7	4.3	5.1	31.5	30.6	32.4
**Antibodies**													
N-AB positive	9084	30.1	29.2	31.0	3.2	2.8	3.6	7.1	6.6	7.7	59.6	58.6	60.6
N-AB negative	11646	86.1	85.5	86.7	1.3	1.1	1.5	2.3	2.0	2.5	10.3	9.8	10.9
S-AB positive	20038	61.5	60.8	62.1	2.2	2.0	2.4	4.4	4.2	4.7	31.9	31.3	32.6
S-AB negative	678	64.5	60.9	68.0	1.3	0.5	2.2	3.4	2.0	4.8	30.8	27.4	34.3
**Comorbidities***													
Cancer (current or treated in the last year)	1091	69.8	67.0	72.5	2.2	1.3	3.1	3.5	2.4	4.6	24.6	22.0	27.1
Cardiovascular disease	2001	73.0	71.1	75.0	2.3	1.6	3.0	3.2	2.4	3.9	21.5	19.7	23.3
Diabetes	2028	72.4	70.4	74.3	1.5	1.0	2.0	4.4	3.5	5.3	21.7	19.9	23.5
Hypertension	7055	68.6	67.5	69.7	2.0	1.7	2.3	3.9	3.5	4.4	25.5	24.5	26.5
Immunosuppression	429	70.6	66.3	74.9	2.3	0.9	3.8	3.3	1.6	4.9	23.8	19.8	27.8
Lung disease	2507	63.7	61.8	65.5	2.6	1.9	3.2	4.4	3.6	5.2	29.4	27.7	31.2

*only in the ≥ 18years
